# A Case of Multifocal Sclerosing Angiomatoid Nodular Transformation (SANT) of the Spleen: A Case Report and Literature Review

**DOI:** 10.7759/cureus.92481

**Published:** 2025-09-16

**Authors:** Yurie Ando, Hiroyuki Tanaka, Takashi Goto, Shoji Taniguchi, Rintaro Koga

**Affiliations:** 1 Surgery, Koga General Hospital, Miyazaki, JPN; 2 Pathology, Koga General Hospital, Miyazaki, JPN

**Keywords:** benign splenic tumor, differential diagnosis, laparoscopic splenectomy, multifocal, sclerosing angiomatoid nodular transformation, spleen

## Abstract

Sclerosing angiomatoid nodular transformation (SANT) is a rare, benign vascular lesion of the spleen that usually presents as a solitary mass. Multifocal SANT is extremely uncommon.

We describe a 37-year-old man who was incidentally found to have multiple splenic lesions during a routine health checkup. Contrast-enhanced computed tomography revealed three solid masses, raising concern for primary splenic lymphoma. Laparoscopic splenectomy was performed, and intraoperative frozen section suggested SANT. Histopathological analysis confirmed the diagnosis, showing three distinct vascular components without malignant features.

Although most reported cases of SANT present with a solitary mass, this case highlights a rare multifocal pattern. The lack of a characteristic imaging finding, the spoke-wheel pattern, and the rather small number of reported cases of multiple SANTs made it difficult to differentiate them from malignant tumors.

Multifocal SANT is exceedingly rare and may mimic malignant splenic tumors, complicating preoperative diagnosis. This case underscores the need to include SANT in the differential diagnosis of splenic masses and suggests that partial splenectomy combined with intraoperative frozen section analysis may offer a less invasive management option. Broader recognition of multifocal SANT could help refine diagnostic algorithms and surgical strategies for splenic tumors.

## Introduction

Sclerosing angiomatoid nodular transformation (SANT), first described by Martel et al. in 2004, is a solitary, well-defined, non-encapsulated, non-neoplastic vascular lesion of the spleen. Histologically, it is characterized by three distinct vascular components, capillaries, small veins, and sinusoid-like vessels, within a dense fibrosclerotic stroma [1]. Although its exact pathogenesis remains unclear, most patients are asymptomatic, and the lesion is often discovered incidentally during imaging performed for unrelated reasons [1]. Characteristic imaging findings, such as the “spoke-wheel” pattern seen on contrast-enhanced CT or MRI, may suggest the diagnosis [2]. However, it is often difficult to distinguish SANT from other splenic tumors, such as malignant lymphoma, inflammatory pseudotumor, or hemangioma [3]. Consequently, splenectomy is frequently performed for both diagnostic and therapeutic purposes.

To date, approximately 200 cases have been reported worldwide, of which 41 cases, including this case, have been reported in Japan, accounting for approximately 25% of all cases [3,4]. Only two multifocal cases have been reported in Japan [5], and very few in other countries. Interestingly, SANT has been reported more frequently in Asian populations [4]. In Japan, in particular, many cases are detected incidentally through routine screening programs such as annual health checkups [6]. Differences in healthcare systems may influence the higher frequency of reported cases compared to Western countries. In this report, we present a rare case of multifocal SANT detected by routine health screening and we discuss its clinical significance in the context of previous literature.

## Case presentation

The patient was a 37-year-old man. He underwent abdominal ultrasonography during a routine checkup one year earlier, which showed no abnormalities. However, during the health checkup this year, his examination revealed splenic tumors, and he was referred to our hospital for further evaluation. His past medical history included infantile asthma and atopic dermatitis. Laboratory findings revealed mild elevation of liver enzymes, while renal function and electrolyte levels were within normal limits. Inflammatory markers were slightly increased. Complete blood count and coagulation profile were unremarkable. Immunoglobulin levels were generally within normal ranges, except for a mild increase in IgA. Tumor markers were within normal ranges. The detailed laboratory findings are summarized in Table 1.

**Table 1:** Laboratory findings VCA, viral capsid antigen; FA, fluorescent antibody.

Test name (unit)	Observed value	Reference range
Total protein (g/dL)	7.6	6.6-8.1
Aspartate aminotransferase, AST (U/L)	47	13-30
Alanine aminotransferase, ALT (U/L)	94	10-42
Lactate dehydrogenase, LDH (U/L)	169	124-222
Gamma-glutamyl transpeptidase, γ-GTP (U/L)	52	13-64
Total bilirubin (mg/dL)	0.76	0.4-1.5
Blood urea nitrogen, BUN (mg/dL)	13.5	8.0-20.0
Creatinine (mg/dL)	0.79	0.75-1.07
Sodium (mEq/L)	141	138-145
Chloride (mEq/L)	102	101-108
Potassium (mEq/L)	4.2	3.6-4.8
C-reactive protein, CRP (mg/dL)	0.59	<0.14
White blood cell count, WBC (/μL)	6,400	3,300-8,600
Hemoglobin, Hb (g/dL)	16	13.7-16.8
Platelet count, Plt (×10³/μL)	298	158-348
Prothrombin time, PT (sec)	13.6	10.0-13.5
Activated partial thromboplastin time, APTT (sec)	32.7	26.5-40.0
Immunoglobulin A, IgA (mg/dL)	426	93-393
Immunoglobulin G, IgG (mg/dL)	1,128	861-1747
Immunoglobulin M, IgM (mg/dL)	157	33-183
Immunoglobulin G4, IgG4 (mg/dL)	14.5	11-121
Soluble interleukin-2 receptor, sIL-2R (U/mL)	305	122-496
Carcinoembryonic antigen, CEA (ng/mL)	0.6	<4.0
Carbohydrate antigen 19-9, CA19-9 (U/mL)	10	<37
Epstein–Barr virus viral capsid antigen IgG, EBV VCA IgG (titer)	80	<10
Epstein–Barr virus early antigen diffuse/restricted IgG, EBV EA-DR IgG FA (titer)	<10	<10

**Figure 1:**
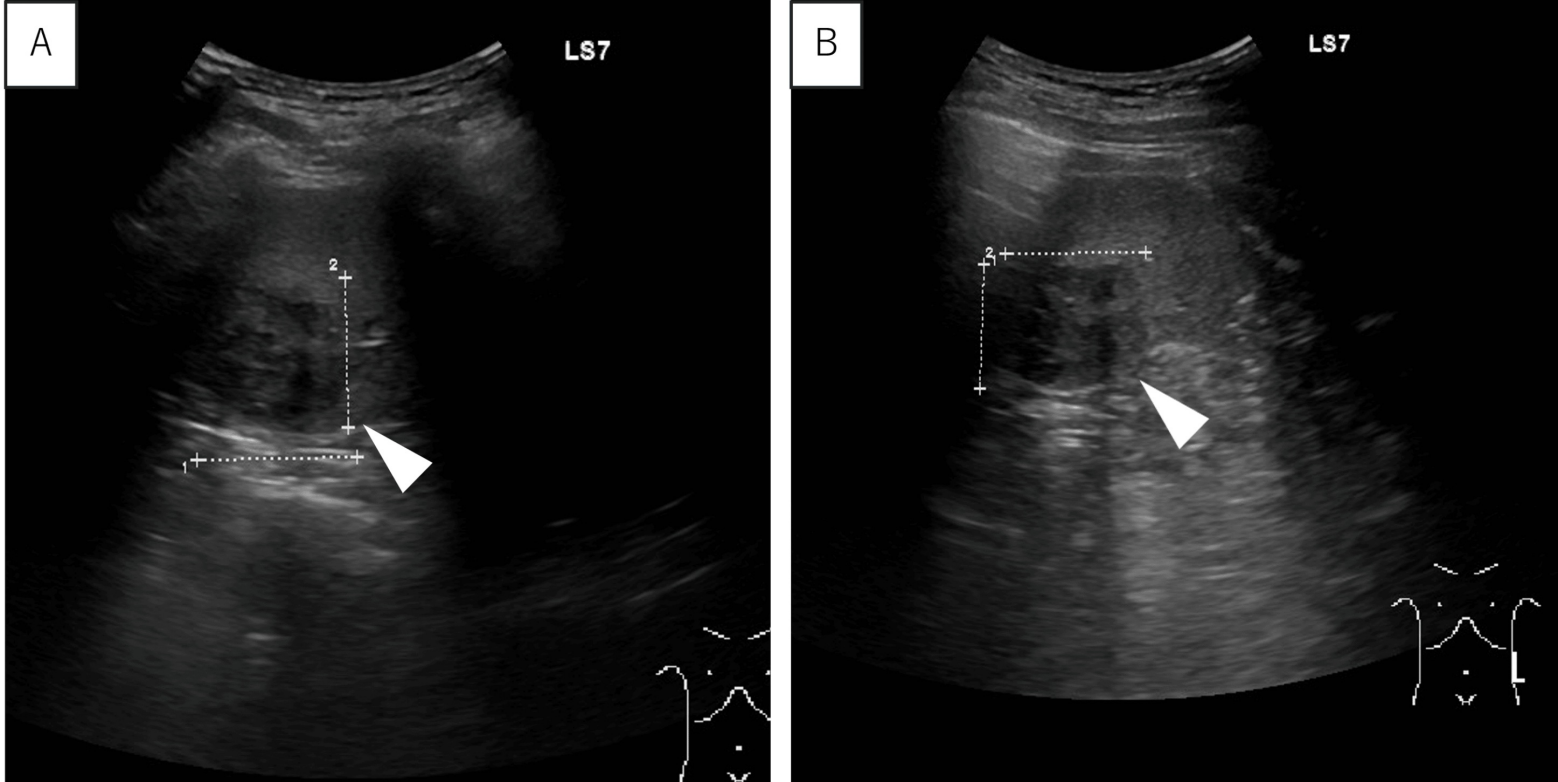
Abdominal ultrasonography Abdominal ultrasonography showing well-circumscribed hypoechoic masses in the spleen. A: Transverse view, B: Longitudinal view

Abdominal ultrasonography revealed well-circumscribed hypoechoic masses in the spleen (Figure 1).

**Figure 2:**
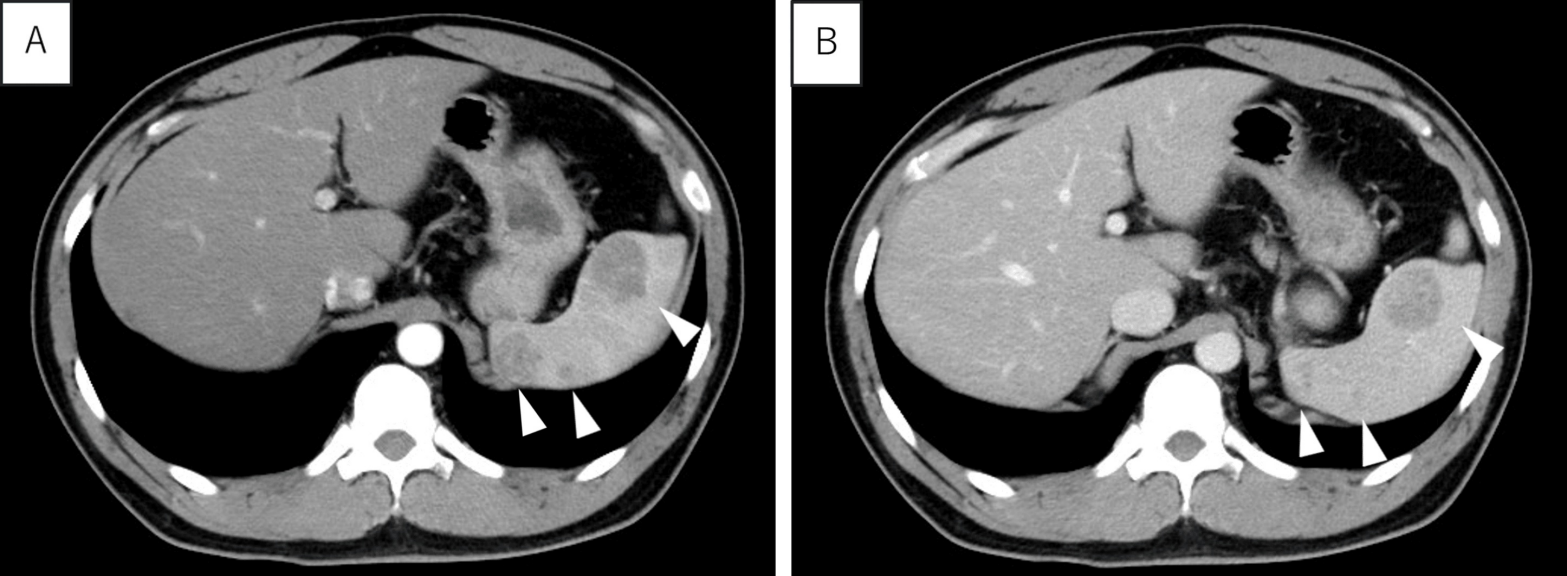
Contrast-enhanced CT CT showed three masses measuring 3.5 cm x 2.7 cm in the hilum, 2.3 cm x 2.1 cm in the upper pole, and 0.5 cm x 0.5 cm, with clear boundaries and poor contrast in the early phase (A) and late phase (B).

Contrast-enhanced CT demonstrated three masses measuring 3.5 cm x 2.7 cm (hilum), 2.3 cm x 2.1 cm (upper pole), and 0.5 cm x 0.5 cm (upper pole), with well-defined margins and minimal enhancement in both arterial and delayed phases (Figure 2). No signs of metastasis, enlarged lymph nodes, or ascites were observed.

Despite normal tumor makers and soluble interleukin-2 (sIL-2) receptor levels, the multifocal nature and absence of findings on the prior ultrasonography raised concern for primary splenic lymphoma. Laparoscopic splenectomy was performed (operation time: 2 hours 24 minutes, blood loss: 10 ml). As the frozen section diagnosis was SANT, lymph node dissection was not performed.

The postoperative recovery was uneventful: oral intake was resumed on day 3, the drain was removed on day 5, and the patient was discharged without complications on day 9. 

**Figure 3:**
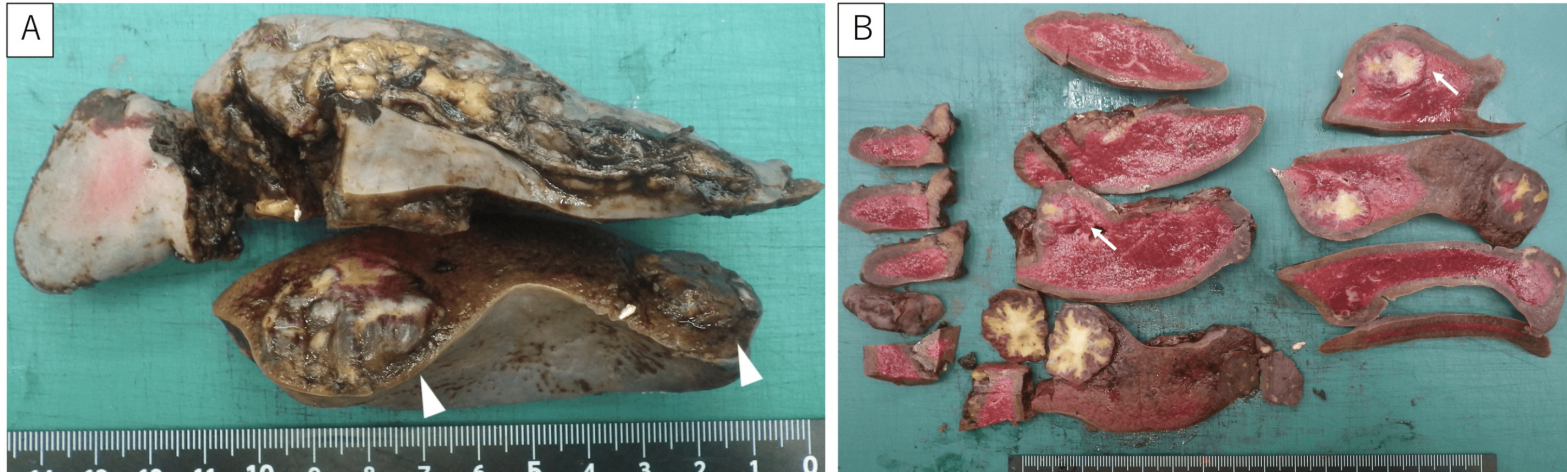
Macroscopic findings (A) Cut surface showing two well-circumscribed nodular lesions: 2.5 cm × 2 cm on the superior pole side and 3.5 cm × 2.7 cm on the splenic portal side. The two lesions on the superior pole side identified on CT were one lesion, both macroscopically and microscopically. (B) Sectioned specimen: The white arrow indicates the area corresponding to the histological findings shown in Figure 4.

Macroscopic findings revealed a spleen measuring about 13 cm x 6 cm. Two lesions were identified: one 2.5 cm x 2 cm at the upper pole and one 3.5 cm x 2.7 cm lesion at the hilum. Although three lesions had been observed radiologically, two lesions identified on CT corresponded to a single lesion on macroscopic and histologic examination (Figure 3).

Both lesions consisted of well-circumscribed nodular areas composed of individual and confluent vascular/ angiomatoid nodules with fibrosclerotic stroma with hematoxylin-eosin stain. They demonstrated a mixture of slit-like vascular spaces lined by plump endothelial cells and pericytes with extravasated erythrocytes.

**Figure 4:**
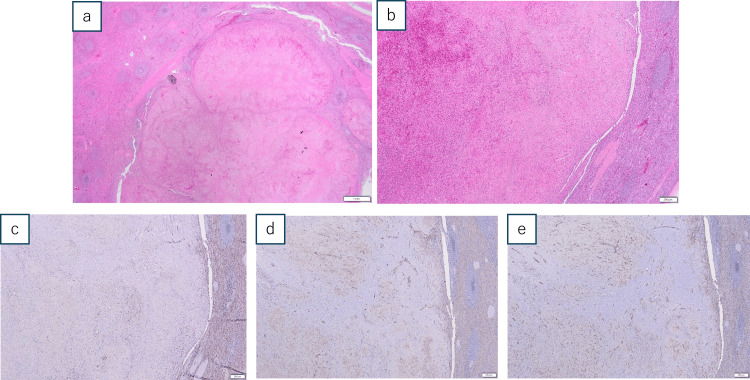
Microscopic findings Hematoxylin-eosin staining (a,b) revealed a nodular lesion septated by fibrosclerotic stroma with a slit-like vascular lumen lined by pump endothelial and pericytes with extravasated erythrocytes (a: 1mm, b: 200μm). From the immunohistochemical study (c: CD8, d: CD31, e: CD34), three different types of vessels within the nodules were identified: (CD8-, CD31+, CD34+), small vessels (CD8-, CD31+, CD34-), and sinusoid-like vessels (CD8+, CD31+, CD34-). They are indicated by white arrows in Figure 3B.

Three types of vessels were identified with the nodules: (CD8-, CD31+, CD34+), small vessels (CD8-, CD31+, CD34-), and sinusoid-like vessels (CD8+, CD31+, CD34-) by an immunohistochemical study (Figure 4). No evidence of malignancy was observed, and final diagnosis of SANT was made.

## Discussion

SANT is a solitary, well-defined, borderline non-neoplastic vascular lesion lacking a capsule, first described by Martel et al. in 2004 [1]. Histologically, it is characterized by the presence of three types of vascular components within the fibrous stroma: capillaries (CD34+, CD8-, CD31+), small veins (CD34-, CD8-, CD31+), and sinusoid-like veins (CD34-, CD8+, CD31+) [1]. The pathogenesis remains unclear, although it has been attributed to microvascular damage and reactive inflammatory processes [1,7], as well as to IgG4-related diseases [8] and Epstein-Barr virus (EBV) [8,9]. Clinically, SANT is often asymptomatic and discovered incidentally [1]. Contrast-enhanced CT and MRI are characterized by a “spoke-wheel” pattern that progressively contrasts from the tumor margins toward the center but is not always depicted and therefore not diagnostic [2,3]. In our case, there was no change in enhancement between the early and delayed phases, and the typical spoke-wheel appearance was absent due to heterogeneous internal contrast and hypovascular areas.

An immunohistochemical study is essential for definitive diagnosis. Sonography-guided percutaneous needle biopsy for splenic lesions has been reported to have a diagnostic rate of over 80% with few complications [10,11]. However, only two cases of SANT have been definitively diagnosed by preoperative biopsy, reported by Gutzeit et al. [12] and Van den Eede et al. [13]. There have been no such reports from Japan. This may be due to the difficulty in distinguishing benign from malignant tumors radiologically, and there is a possibility of tumor seeding by needle biopsy if the lesion is malignant. Therefore, the decision to perform a preoperative biopsy must be made carefully.

The major differential diagnoses include primary splenic malignant lymphoma, hemangioma, and inflammatory pseudotumors. In particular, malignant lymphoma, the most important differential diagnosis, often presents with splenomegaly, hypodense lesions on CT, and iso- to hypointensity on T2-weighted MRI [14]. However, differentiation based on imaging alone remains difficult, and therefore splenectomy is often performed for diagnostic treatment [1,5].

In particular, multifocal SANT presents even greater diagnostic and therapeutic challenges compared to solitary cases. The presence of multiple splenic lesions strongly raises suspicion for malignant lymphoma, making preoperative recognition of SANT extremely difficult. From a surgical perspective, while partial splenectomy has been proposed in solitary cases to preserve splenic function and reduce the risk of overwhelming postsplenectomy infection (OPSI), this approach is generally not feasible in multifocal disease. Given the extreme difficulty in differentiating multifocal SANT from malignancy, total splenectomy remains the most practical therapeutic option.

Even when a benign tumor is suspected and the patient is monitored, many tumors increase in size over time, and malignancy cannot be ruled out, often resulting in surgery in most cases [15]. In some cases, partial splenectomy may be performed to avoid the risk of OPSI, a serious complication [16,17]. When malignancy can be ruled out intraoperatively by frozen section analysis, partial splenectomy is a valid option. Although we performed a total splenectomy, partial resection followed by frozen section analysis might have provided a less invasive surgical approach.

A search for “sclerosing angiomatoid nodular transformation” on PubMed revealed that approximately 200 cases of SANT have been reported to date [4,14], most of which are solitary lesions. A PubMed search using the term “SANT, multiple” yielded no hits. In Japan, 41 cases have been reported so far, with only one case reported prior to ours by Hirota et al. in 2014 [5]. This suggests an incidence of only 0.04% for multifocal cases in Japan. Interestingly, however, recent data from China by Xiang et al. (2025) reported that approximately 10% of SANT cases were multifocal [4], suggesting that solitary and multifocal SANT may be more common in Asian countries.

In Japan, many SANT cases are detected incidentally through routine physical examinations, including abdominal ultrasonography, an approach less common in Western countries. These differences in healthcare systems and diagnostic practices may contribute to the relatively high number of reported cases from Japan and other Asian countries. According to the Ministry of Health, Labor and Welfare in Japan, national health check-ups are conducted annually, with a participation rate exceeding 80% [6]. In major urban areas in China, similar programs have been reported with uptake rates of 60-70%, particularly among employed individuals [18]. Such differences in routine screening may contribute to increased frequency of incidentally detected rare splenic lesions such as SANT in Asian countries.

However, definitive geographic or ethnic predisposition has not yet been identified. Large-scale epidemiologic studies are needed to determine whether these differences reflect true regional variation or are the result of reporting and diagnostic biases. This case contributes valuable information to the literature on multifocal SANT. As more cases are reported, our understanding of its epidemiology, etiology, and optimal management is expected to improve.

## Conclusions

This case represents an extremely rare instance of multifocal SANT. Multifocal lesions are more difficult to distinguish from malignant tumors than solitary lesions and present greater diagnostic challenges. Although laparoscopic splenectomy was ultimately performed, the combination of partial resection and intraoperative frozen section analysis may have allowed for a less invasive surgical option. Further accumulation of cases may improve the accuracy of preoperative diagnosis and aid in the development of more tailored treatment strategies.
